# Cellular senescence, geroscience, cancer and beyond

**DOI:** 10.18632/aging.101546

**Published:** 2018-09-07

**Authors:** Francis Rodier, Daohong Zhou, Gerardo Ferbeyre

**Affiliations:** 1 Centre de Recherche du Centre Hospitalier de l’Université de Montréal (CRCHUM), Montreal, QC, Canada; 2 Institut du Cancer de Montréal, Montreal, QC, Canada; 3 Department of Radiology, Radio-Oncology and Nuclear Medicine, Université de Montréal, Montreal, QC, Canada; 4 Department of Pharmacodynamics, College of Pharmacy, University of Florida, Gainesville, FL 32610, USA; 5 Department of Biochemistry and Molecular Medicine, Université de Montréal, Montreal, QC, Canada

**Keywords:** aging, senescence, cancer, age-related diseases, geroscience

More than two hundred scientists gathered in Montreal on July 8, 2018 for the International Cellular Senescence Association (ICSA) Meeting to discuss the biological and medical impact of cellular senescence. The meeting was organized by **Gerardo Ferbeyre** (Université de Montréal, Canada), **Francis Rodier**, (Université de Montréal, Canada) and **Daohong Zhou** (University of Florida, USA). In his welcoming speech, **Dr. Ferbeyre** summarized the key aspects that have attracted so much interest in cellular senescence including its ability to act as a tumor suppressor mechanism but also to promote aging and age-linked diseases (Figure 1). The dual nature of senescence was also highlighted in talks from **Judith Campisi** (Buck Institute for Research on Aging, USA), ICSA president **Manuel Serrano** (IRB, Spain) and NCI director **Ned Sharpless** (National Cancer Institute, USA). **Dr. Sharpless** presented in his keynote lecture how the key senescence gene and tumor suppressor p16INK4A acts as a double-edged sword to regulate aging, health span and cancer incidence.

**Figure 1:**
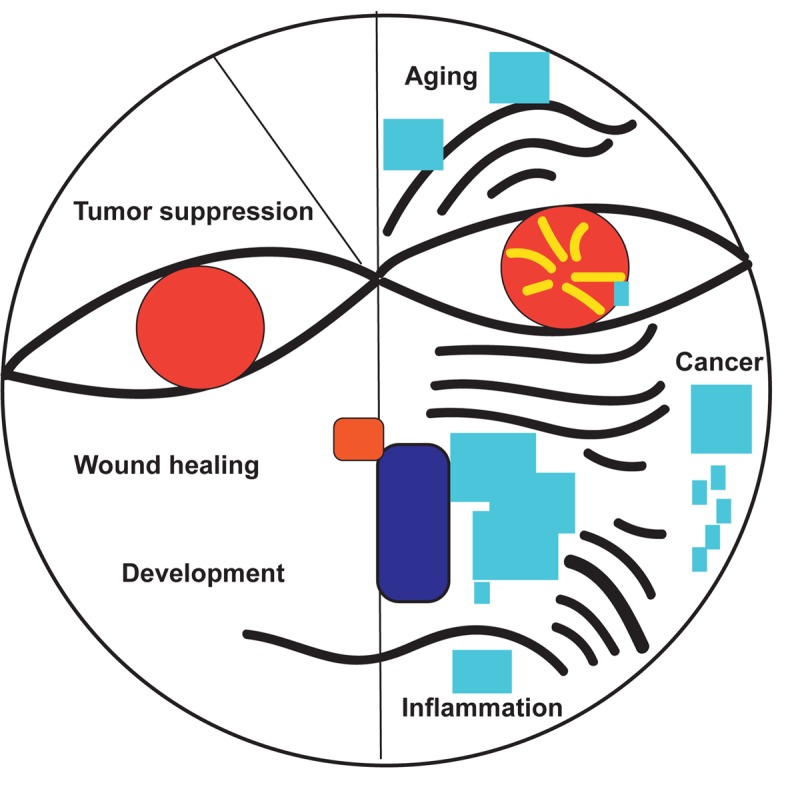
The good and bad of senescence.

## Mechanisms of senescence

**Frederic Lessard** from the Ferbeyre laboratory (Université de Montréal), described the senescence-associated ribosome biogenesis defects and how they are linked to the inhibition of CDK4 via accumulation of ribosome-free RPS14. This work expands the extraribosomal functions of ribosomal proteins, which are now linked to both the p53 and the RB tumor suppressor pathways [1]. **Guadalupe-Elizabeth Jimenez** (CINVESTAV, Mexico) showed an intriguing connection between B-distroglycan and the nucleolus in senescent cells. **Jiri Bartek** (Copenhagen, Denmark), delivered the EMBO Keynote lecture summarizing the role of replication stress and the DNA damage response in cellular senescence [2]. Recent work from his laboratory showed that PARP inhibition leads to accelerated replication fork progression causing replication stress in tumor cells [3]. **Fred Dick** (Western University, Canada), presented novel roles of RB suppressing the expression of satellite repeats and regulating chromosome condensation [4]. **Karl Riabowol** (University of Calgary, Canada) presented a link between senescence and endocytosis via RB in cells that express the ING1a epigenetic regulator. Jesus Gil (MRC, UK) and **Mathieu Deschênes** (Université de Sherbrooke, Canada) presented data linking the control of alternative splicing to cellular senescence.

Several talks described a clear connection between senescence and metabolism. **Xiaolu Yang** (University of Pennsylvania, USA) explained how p53 represses major forms of NADPH generation via distinct mechanisms, clarifying why reactive oxygen species and oxidative damage accumulate in senescent cells. Talks form **Eiji Hara** (Osaka University, Japan), **Maria Grazia Vizioli** (Beatson Institute, UK) and **Andrea Ablasser** (EPFL, Switzerland), provided links between DNA metabolism, the activation of the cGAS-STING pathway and the regulation of the senescence-associated secretory phenotype (SASP). **Masashi Narita** (Cancer Research UK Cambridge Institute, UK) summarized the dual role of autophagy in cancer and presented a mouse model where conditional inactivation of ATG5 altered cancer susceptibility. **Frédérick A. Mallette** (Université de Montréal) presented an intriguing connection between cholesterol 25-hydroxylase (CH25H) and cellular senescence. In a mouse model of retinopathy [5], *Ch25h* mRNA levels increased and correlated with induction of features of cellular senescence in the retina. Perhaps, related to this discovery, **Dr. Hara** presented evidence that another cholesterol metabolite, deoxycholic acid (DCA), induced senescence in liver cells; interestingly, DCA is produced by the gut microbiota during obesity in mice and facilitates the development of hepatocellular carcinoma [6].

**Andrei Gudkov** (Roswell Park Comp. Cancer Center, USA) challenged several existing views of senescence from the analysis of the transcriptome of senescent cells and cells arrested by contact inhibition. Using principal component analysis he proposed that the gene expression pattern of senescent cells is a superposition of two components: one, which depends on the time cells are in culture without dividing, and the other one, which depends on the type of treatment. **Oliver Bishof** (Pasteur Institute, France) also presented a kinetic transcriptome analysis of cells rendered senescent by oncogenic ras. He described an intriguing dynamic gene expression program driven by a hierarchical network of transcription factors reminiscent of a developmental process. Along the same lines, **Judith Campisi** discussed how multiple functional senescence-associated phenotypes are interlaced and dynamic in the senescence program, particularly in different cells types and tissue contexts, and presented a new effort to globally identify senescent cells surface molecules.

## Senescence and cancer

A number of talks solidified the view that senescence is a powerful tumor suppressor mechanism. **Scott Lowe** (Sloan Kettering, USA), who together with **Manuel Serrano** discovered the process of oncogene induced senescence, presented data explaining how cancer cells can be forced into an RB-dependent but p53-independent senescence using a combination of a MEK inhibitor with the CDK4 inhibitor palbociclib. Intriguingly, this treatment also engages NK cells to kill senescent tumor cells, underscoring the view that pro-senescence cancer therapies benefit from immune surveillance mechanisms. Regarding immune clearance of senescent cells, **Christian Beausejour** (Université de Montréal) revealed his efforts to develop novel humanized mouse models to better mimic interactions between senescent cells and immune cells. He showed that senescent human cells are not always immunogenic in this context, suggesting strong context-dependent effects and the importance of using relevant models. The importance of the elimination of senescent cells after treatment was highlighted in several talks. **Clemens Schmitt** (Charité, University Medical Center, Germany) was the first keynote speaker of the meeting. He presented evidence for an underlying stemness gene expression signature in senescent cells. Cells that managed to escape from senescence, take advantage of this stemness program to form aggressive cancers [7]. **Konstantinos Evangelou** (University of Athens, Greece) also focused on cells that escaped from senescence as demonstrated with a novel senescence biomarker (SenTraGor^TM^) [8]. Escaped cells harbor an altered genomic landscape, due to Rad52-dependent error prone DNA repair and exhibit aggressive features including increased resistance to chemotherapy [9,10]. **Corinne Abbadie** (University of Lille, France) characterized the senescence response to irradiation in fibroblasts at the margin of an irradiated field. She explained that these cells accumulate single- stranded breaks but not double-stranded DNA breaks and they were able to escape from senescence giving rise to a progeny of mutated cells. From a more clinical angle, **Francis Rodier** explored the occurrence of therapy-induced senescence in human cancer patients particularly demonstrating that senescence occurs in response to ovarian cancer chemotherapy. Interestingly, the presence of senescence hallmarks in treated ovarian tumors predicted beneficial outcomes in patients, suggesting that senescence biomarkers could help inform cancer treatment strategies and that senescence becomes a target for pharmacological manipulation in human ovarian cancer therapy. **Olivier Coqueret** (Université d’Angers, France) presented data on TSP1 acting as a cytokine that prevents escape from senescence and described how its receptor CD47 regulates escape from the senescent arrest. **Dr. Serrano** presented data indicating that palbociclib, a CDK4 inhibitor approved for the treatment of some cancers, accumulates in lysosomes and this prolongs its biochemical effects explaining the induction of senescence even after a short period of exposure to the drug. The storage of palbociclib into lysosomes also accounts for its delayed and long-term release to the external milieu and the efficient induction of paracrine senescence, which in this case is mostly due to the drug and not to the SASP. **Marco Demaria** (European Research Institute for the Biology of Ageing, Netherlands) further expanded on the use of CDKi to induce a potentially less inflammatory senescence response in normal and cancer cells, as he demonstrated the lack of a typical SASP in this context. He suggested that this could be beneficial in some therapeutic contexts, but harmful in others, as SASP-less senescence might alter normal immune clearance of senescent cancer cells. In summary, a theme emerged that any therapy that aims to induce or reinforce senescence in tumors should also be combined with strategies to prevent escape from senescence or to stimulate the elimination of senescent cells.

## Senolysis, aging and age-linked diseases

One of the most exiting trends in senescence research is the concept of senolysis or the specific elimination of senescent cells [11]. **Jan Van Deursen** (Mayo Clinic, USA) presented recent evidence that the elimination of senescent cells can induce regression of advanced atherosclerosis without any detectable side effects. **Jennifer Hartt Elisseeff** (Johns Hopkins, USA) showed that clearance of senescent cells using senolytics attenuates osteoarthritis development. The connection between senescent cells and immune responses to injury and repair was presented. **Darren Baker** (Mayo Clinic, USA) presented experimental evidence that senescent cells promote neurodegeneration in mutant tau mice [12] and their elimination attenuates disease. **James Kirkland** (Mayo Clinic, USA), showed that transplanting senescent cells to young mice caused frailty, diabetes and osteoporosis accelerating death from all causes. A cocktail of quercetin and dasatinib, a SRC-family kinase inhibitor, can kill senescent cells and revert their pathological effects both in senescent-cells transplanted young mice or in naturally aged mice, extending median life span up to 36% [13]. **Salvador Macip** (University of Leicester, UK) found another kinase, BTK, which activates the tumor suppressor p53 inducing senescence [14]. Ibrutinib, a clinically approved inhibitor for this kinase increased life span in flies and in a mouse model of progeria. **Irina Conboy** (UC Berkeley, USA) used parabiosis to demonstrate the presence of factors in the serum of old mice that can induce senescence in young mice suggesting that some senescent cells in vivo may originate from extrinsic factors. She also presented interesting data on enhanced myogenesis and reduced liver adiposity, but no improvement in hippocampal neurogenesis in the old 3MR mice, when p16-high cells were experimentally ablated. **Albert Davalos** (Buck Institute for Research on Aging) followed-up on his earlier discovery that the alarmin HMGB1 is a key regulator of the proinflammatory SASP [15] by showing that HMGB1 can induce paracrine senescence and is involved in aged serum-induced senescence. **Myriam Gorospe,** (NIH, USA) identified proteins expressed at the surface of senescent cells. SCAMP4 was found to favor the SASP [16] and DPP4 was found to allow the selective elimination of senescent cells using anti-DPP4 antibodies [17]. **Mei Wang** (Johns Hopkins University School of Medicine, USA) presented her work showing that mesenchymal stem/progenitor cells (MSPCs) in primary spongiosa of long bone during late puberty undergo a normal programed senescence. MSPC senescence is epigenetically controlled by the polycomb histone methyltransferase Ezh2 and its H3K27me3 mark. Loss of Ezh2-H3K27me3 in young mice leads to premature cellular senescence followed by impaired osteogenesis and bone loss, and antagonizing cellular senescence by manipulating epigenetic factors may be a potential approach to treat pediatric or juvenile osteoporosis. **Maria Almeida** (University of Arkansas for Medical Sciences, USA) discussed the role of senescent osteocytes in age-related bone loss via production of increased levels of RANKL and the therapeutic potential of senolytic agents in preventing and treating osteoporosis by targeting senescent cells in the bones [18]. **Claude LeSaux** (University of California at San Francisco, USA) reported some new interesting findings that eicosanoids such as prostaglandins and leukotrienes may function as new SASP factors and play an important role in the pathogenesis of pulmonary fibrosis. Together these studies show the tremendous potential of senolytics to improve health at old ages.

## Anti-senescence drug discovery

The promise that clearance of senescent cells with a therapeutic agent may prolong the health span and treat age-related diseases stimulates the research in finding new senolytic agents, therapeutic strategies, and delivery methods. **Daohong Zhou** (University of Florida, USA) presented some new development of Bcl-xl-targeted senolytic agents using proteolysis targeting chimera (PROTAC) technology. These Bcl-xl PROTACs that target Bcl-xl to an E3 ligase for ubiquitination and degradation exhibit an improved potency against senescent cells but reduced toxicity to normal cells and platelets compared to navitoclax or ABT-263 and thus have the potential to be developed as a safer senolytic agent. **John Lewis** (Oisin Biotechnologies, USA) described a clinically viable gene therapy consisting of a suicide gene under a senescent cell promoter delivered in vivo with fusogenic lipid nanoparticles (LNPs) to deplete senescent cells. This approach represents a first-in-class therapeutic that targets cells based on transcriptional activity, rather than surface markers or metabolism. **Guangrong Zheng** (University of Florida, USA) identified a dietary natural product, piperlongumine, as a novel senolytic agent. It can selectively kill senescent cells by targeting oxidation resistance 1 (OXR1), an important oxidative stress sensor that regulates the expression of a variety of antioxidant enzymes. His finding may lead to the development of better senolytic agents [19]. **Daniel Munoz-Espin** (University of Cambridge, UK) described the design of a new targeted-drug delivery system to senescent cells using the technology of the encapsulation of drugs with galacto-oligosaccharides because of the high lysosomal β-galactosidase activity of senescent cells. He showed that gal-encapsulated cytotoxic drugs can selectively target senescent cells in a tumor xenograft mouse model to improve tumor regression and toxicity. This senescent cell selective drug delivery method opens new diagnostic and therapeutic applications for senescence-associated disorders. At the end of the meeting **Ned David (**Unity of Biotechnology, USA) delivered a talk summarizing how his company is translating basic research on senescence into clinical trials using several senolytics. Senescence is undoubtedly at the forefront of biomedical research. The next ICSA meeting in Athens 2019 will reveal additional exiting research: stay tuned!

## Organizers

**Figure:**
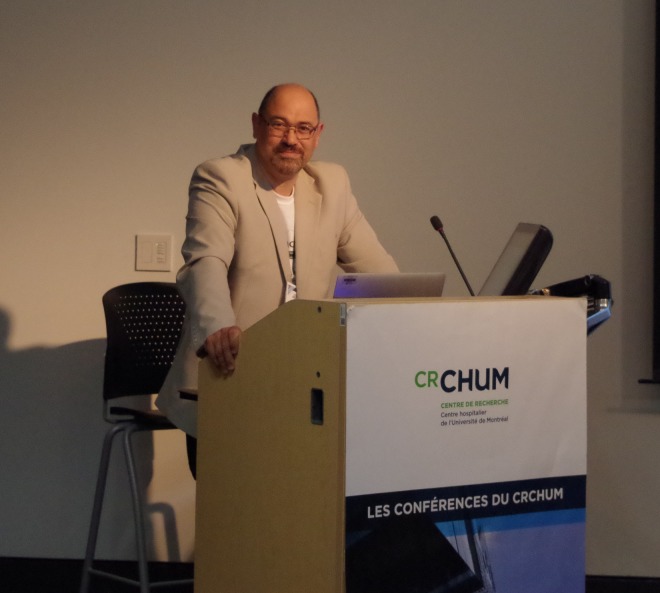
Gerardo Ferbeyre, Université de Montréal and CR-CHUM, Canada

**Figure:**
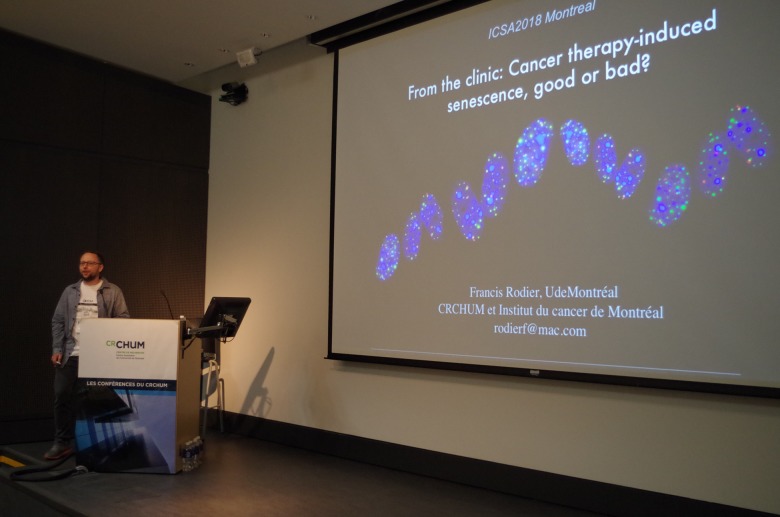
Francis Rodier, CR-CHUM, Université de Montréal, Canada

**Figure:**
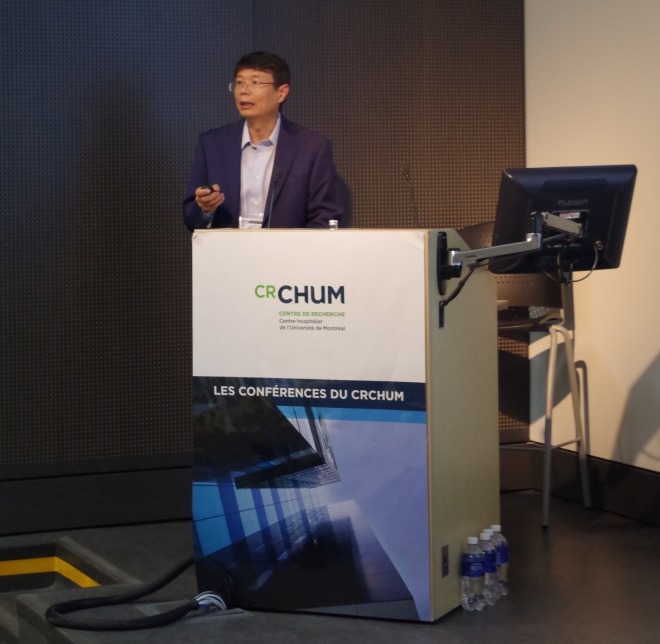
Daohong Zhou, University of Florida, Health Cancer Center, USA

## Keynote Speakers

**Figure:**
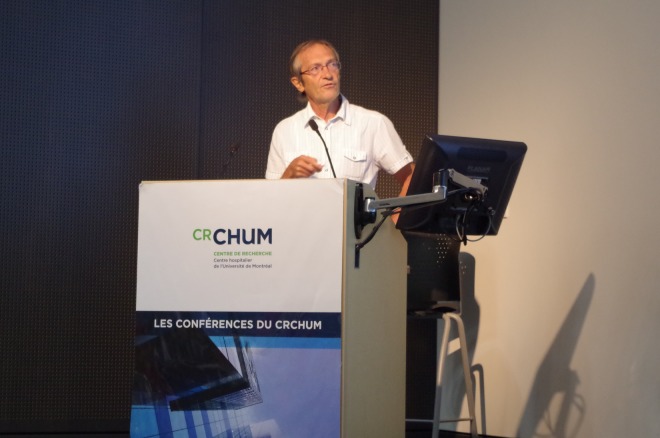
Jiri Bartek, Danish Cancer Society Research Center, Denmark

**Figure:**
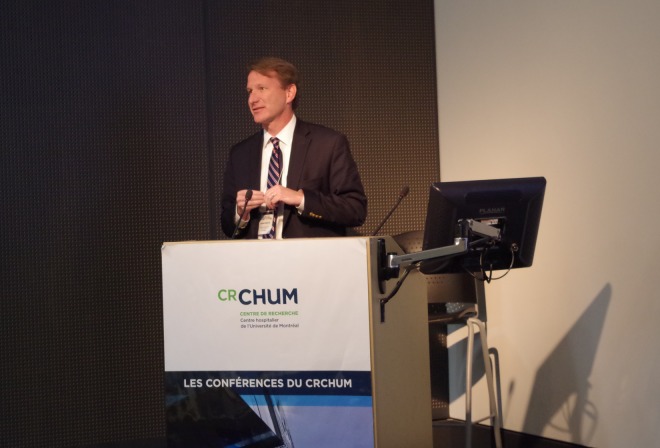
Ned Sharpless, National Cancer Institute, USA

**Figure:**
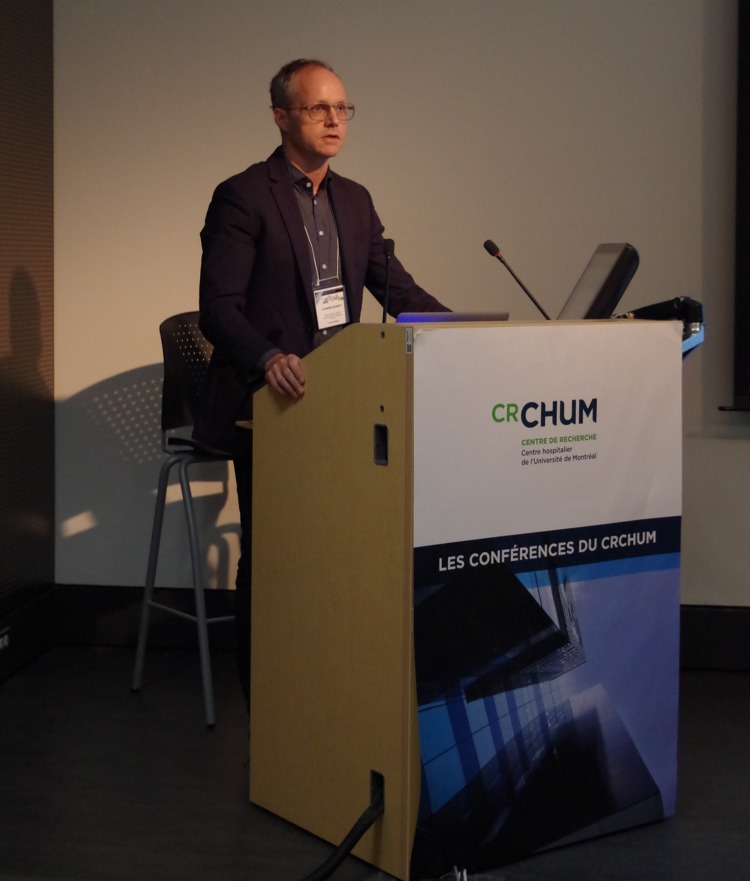
Clemens Schmitt Charité, University Medical Center, Germany

## Invited Speakers

**Figure:**
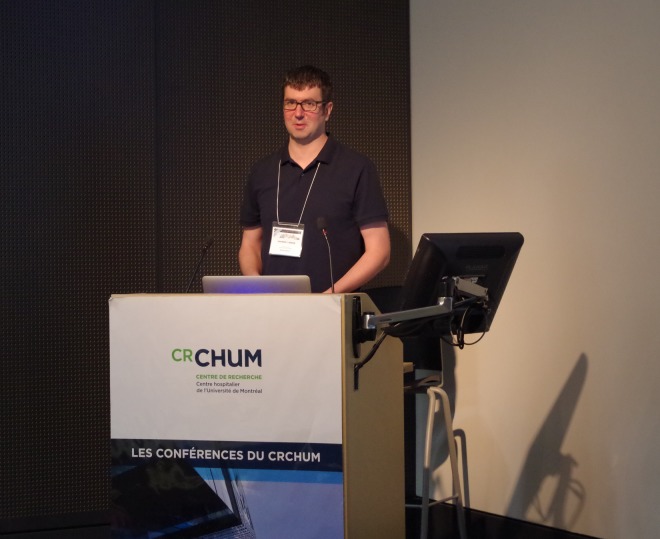
Darren Baker, Mayo Clinic, USA

**Figure:**
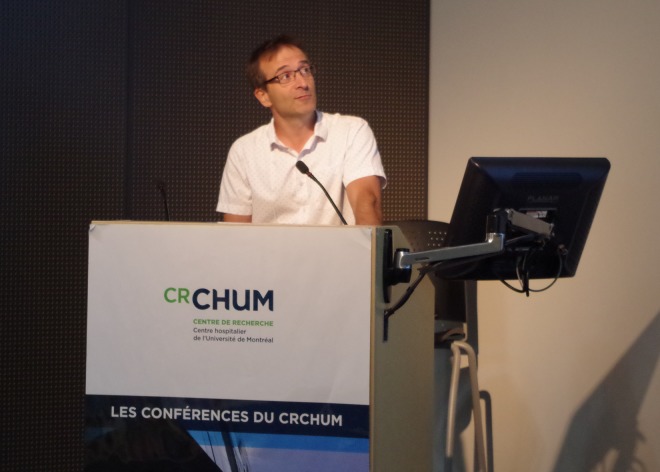
Christian Beauséjour, Université de Montréal, Canada

**Figure:**
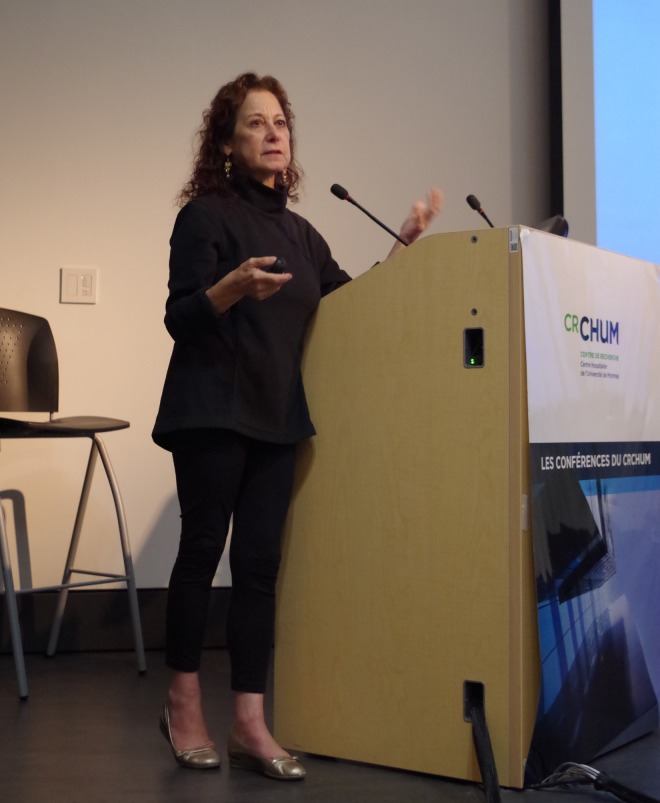
Judith Campisi, Buck Institute for Research on Aging, USA

**Figure:**
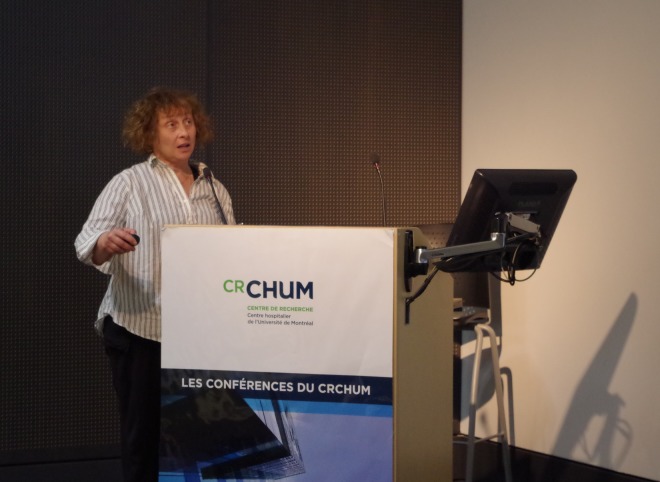
Irina Conboy, University of California at Berkeley, USA

**Figure:**
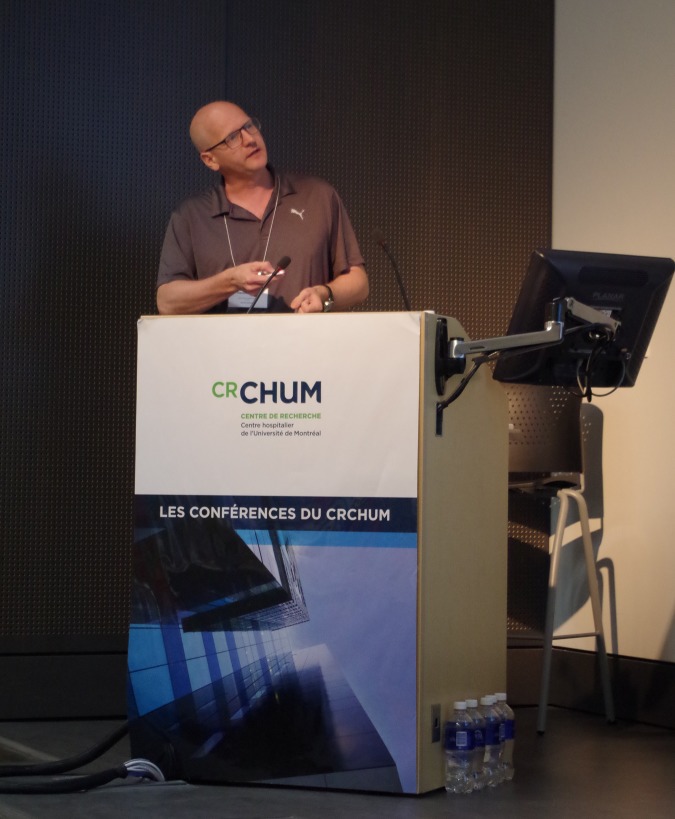
Frederick Dick, Western Canadian University, Canada

**Figure:**
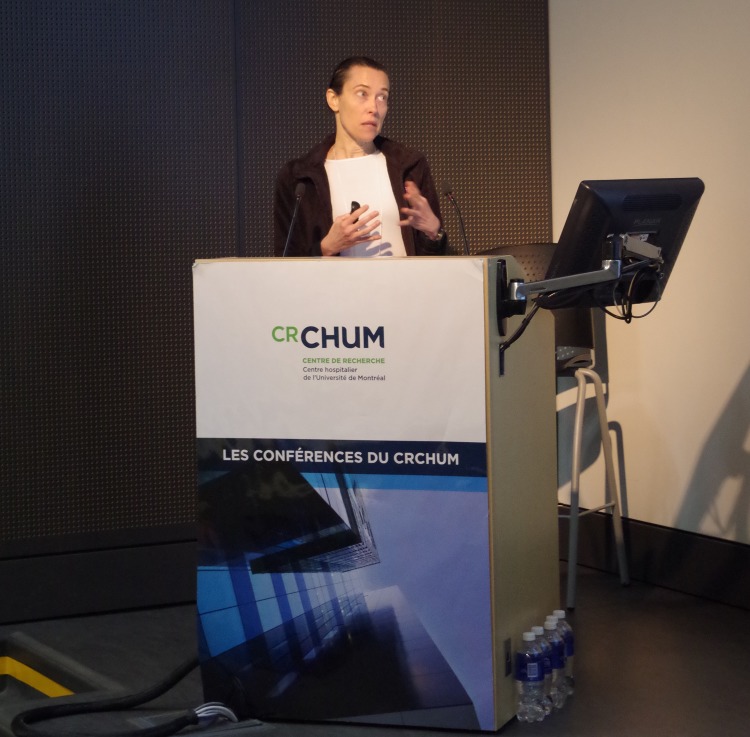
Jennifer Elisseeff, Johns Hopkins Biomedical Engineering, USA

**Figure:**
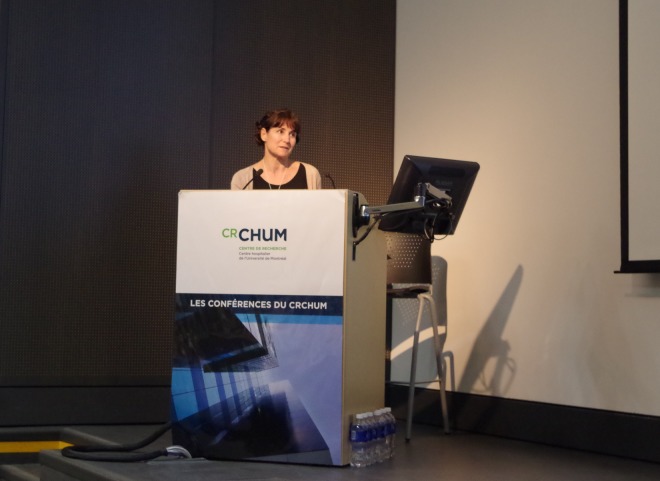
Myriam Gorospe, National Institutes of Health, USA

**Figure:**
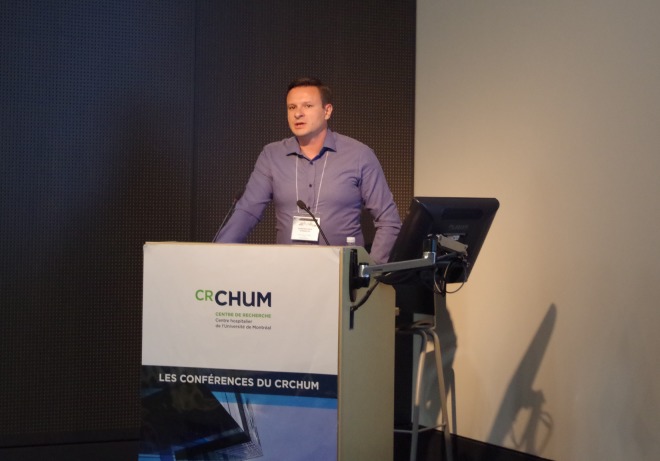
Konstantinos Evangelou, University of Athens, Greece

**Figure:**
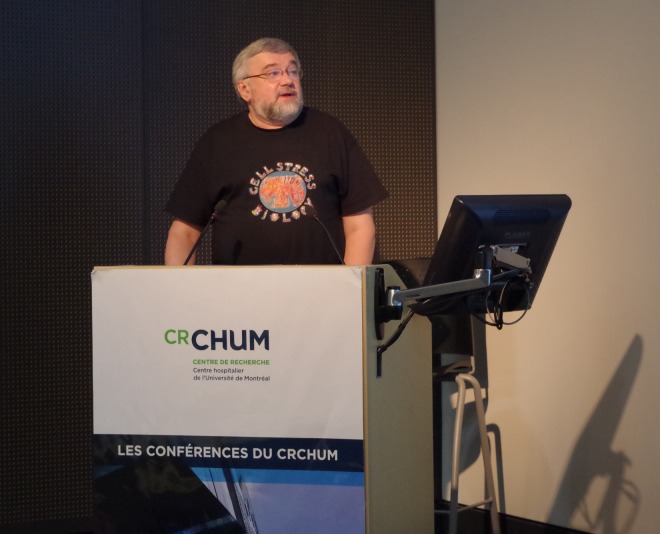
Andrei Gudkov, Roswell Park Cancer Institute, USA

**Figure:**
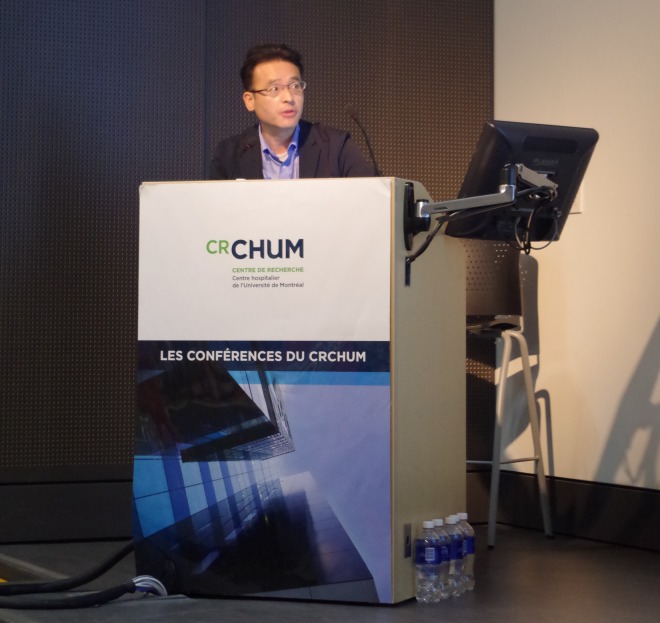
Eiji Hara, Osaka University, Japan

**Figure:**
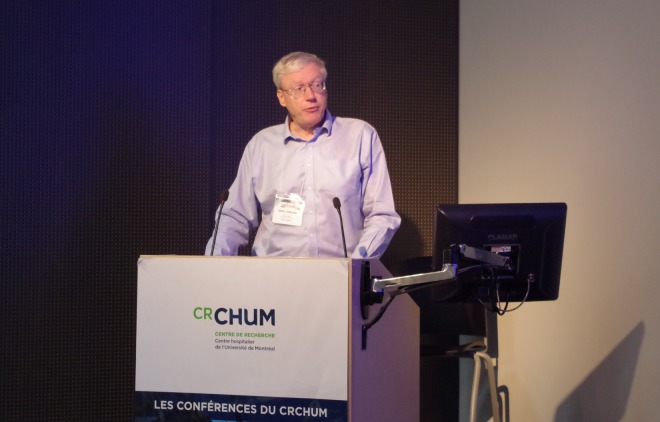
James L. Kirkland, Mayo Clinic USA

**Figure:**
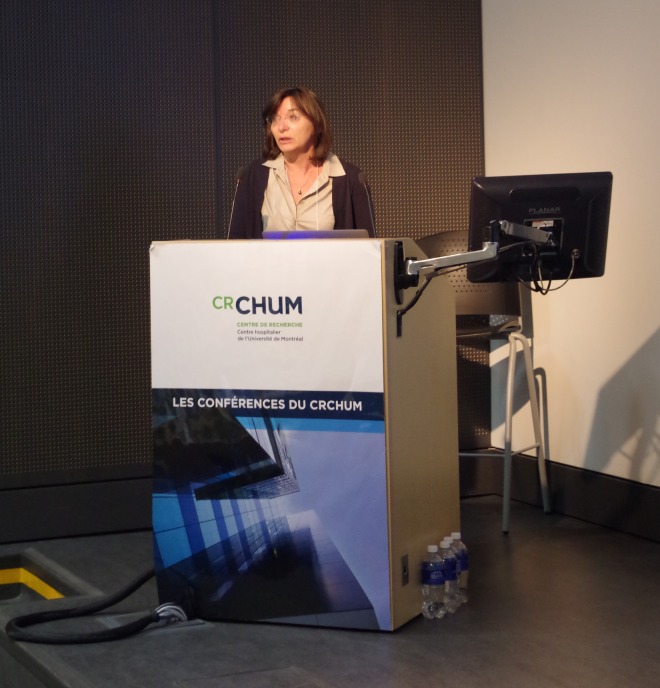
Claude Le Saux, University of California at San Francisco, USA

**Figure:**
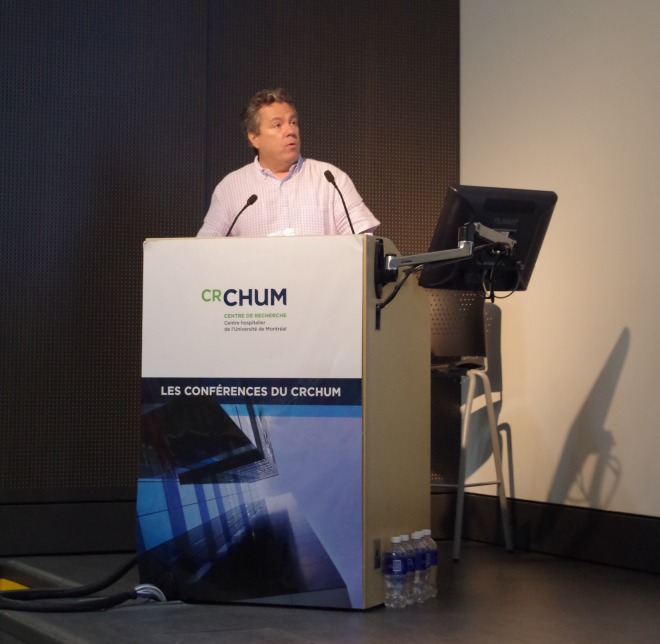
Scott Lowe, Memorial Sloan Kettering Cancer Center, USA

**Figure:**
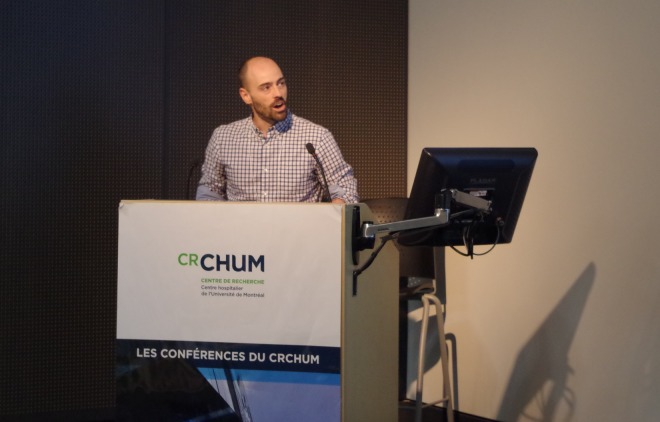
Frédérick A. Mallette, Université de Montréal/Maisonneuve-Rosemont H., Canada

**Figure:**
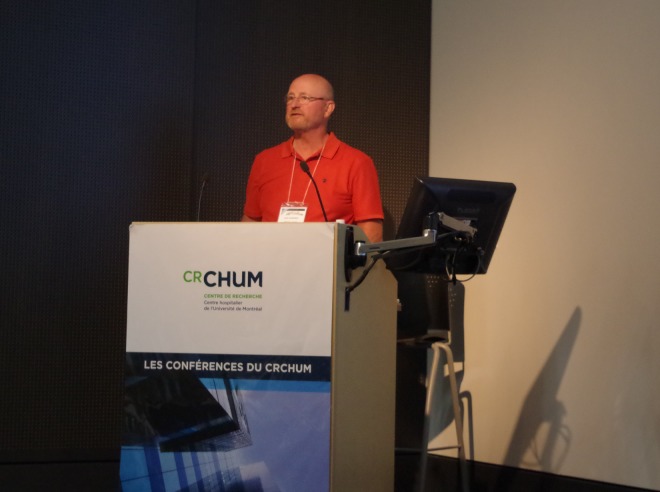
Karl Riabowol, University of Calgary, Canada

**Figure:**
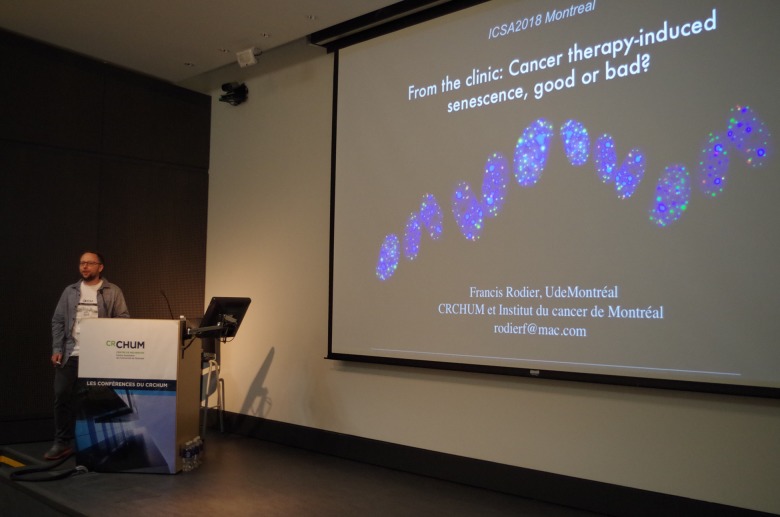
Francis Rodier, CHUM Research Center, Canada

**Figure:**
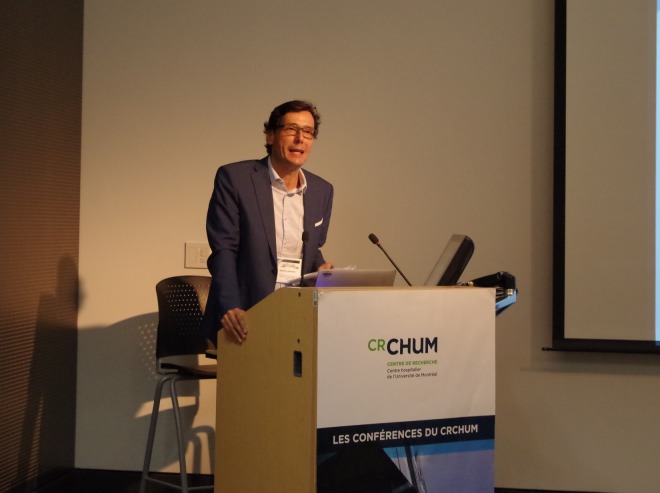
Manuel Serrano, Institute for Research in Biomedicine (IRB), Spain

**Figure:**
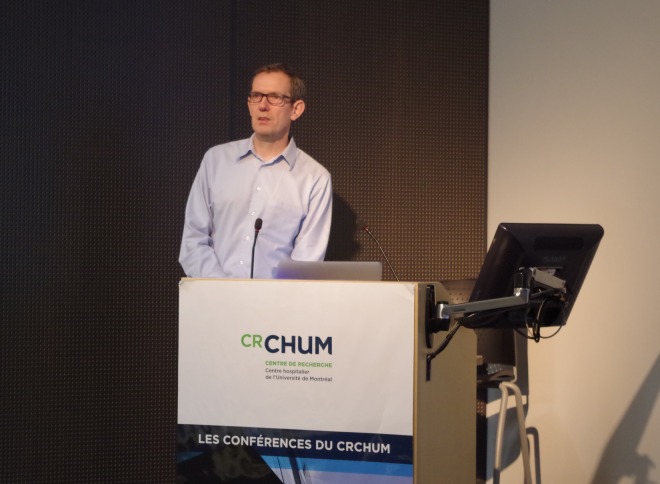
Jan van Deursen, Mayo Clinic, USA

**Figure:**
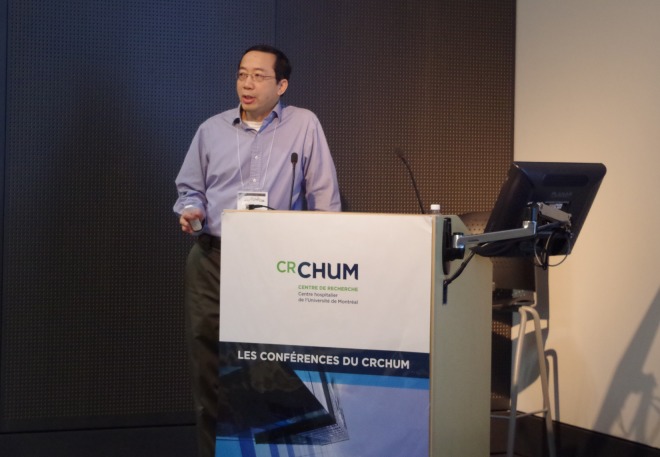
Xiaolu Yang, University of Pennsylvania, USA

**Figure:**
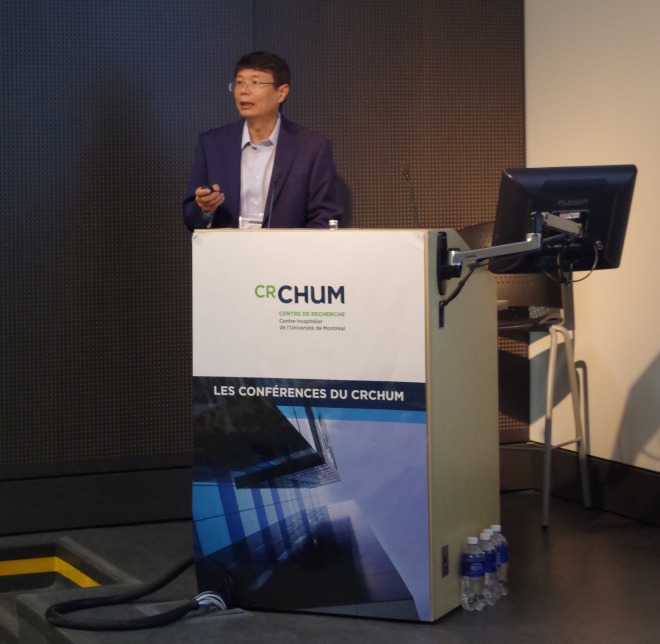
Daohong Zhou, University of Florida, Health Cancer Center, USA

## Poster Sessions

**Figure:**
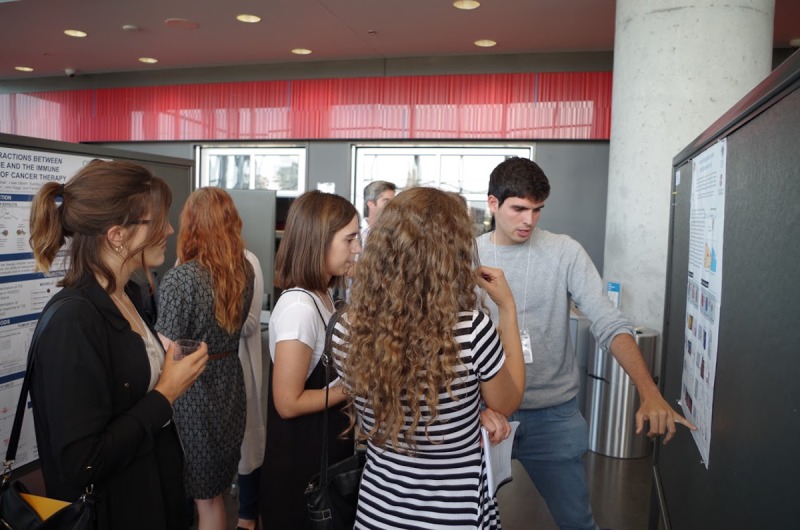
Poster Session Photo 1

**Figure:**
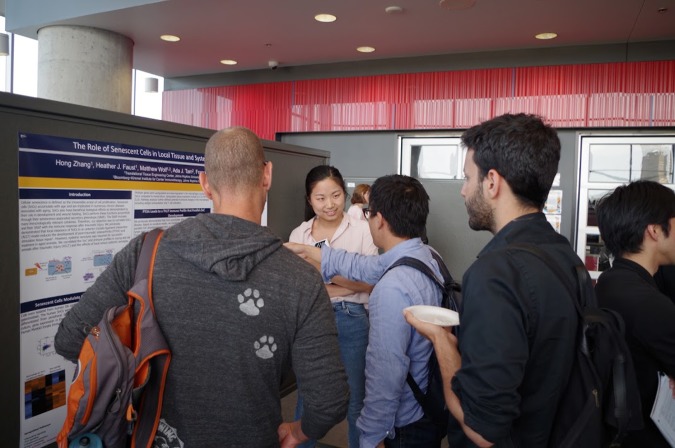
Poster Session Photo 2

**Figure:**
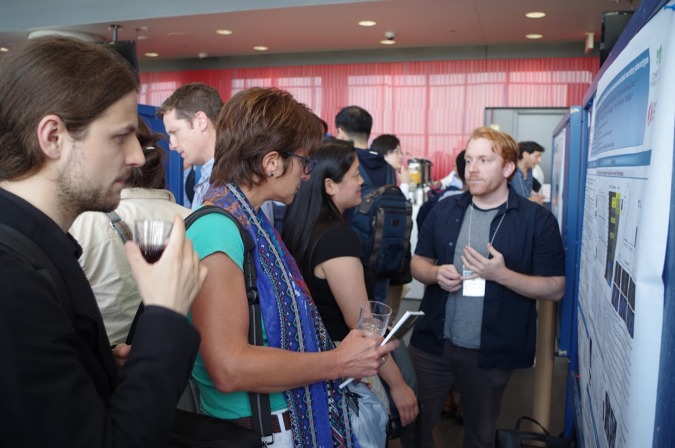
Poster Session Photo 3

**Figure:**
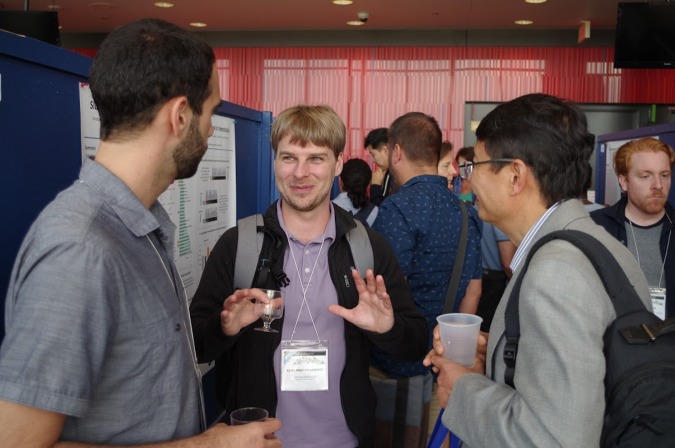
Poster Session Photo 4

**Figure:**
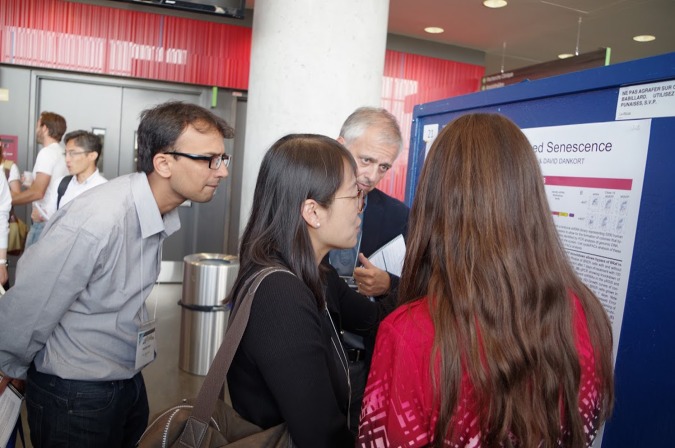
Poster Session Photo 5

**Figure:**
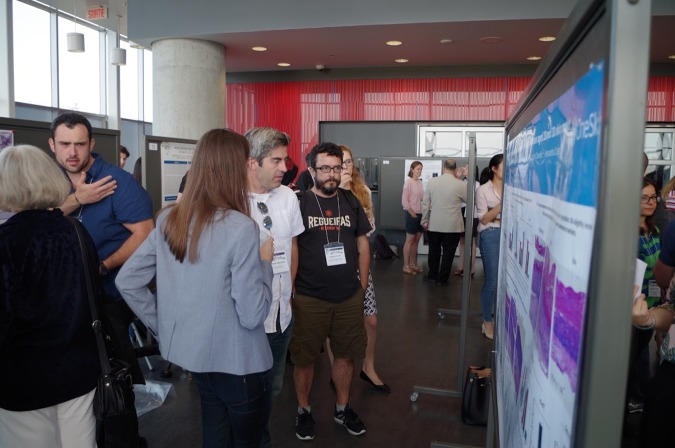
Poster Session Photo 6

**Figure:**
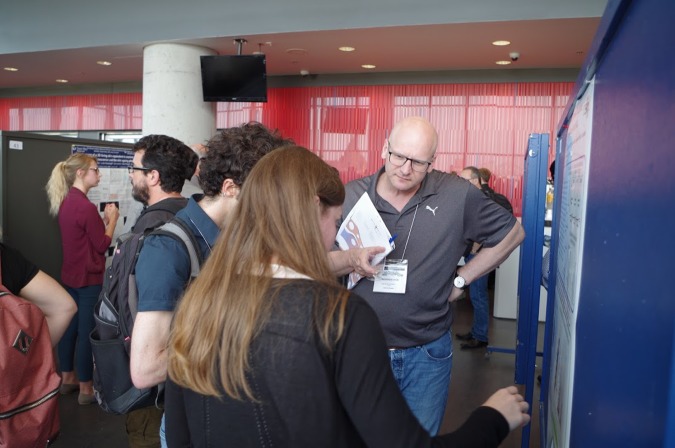
Poster Session Photo 7

**Figure:**
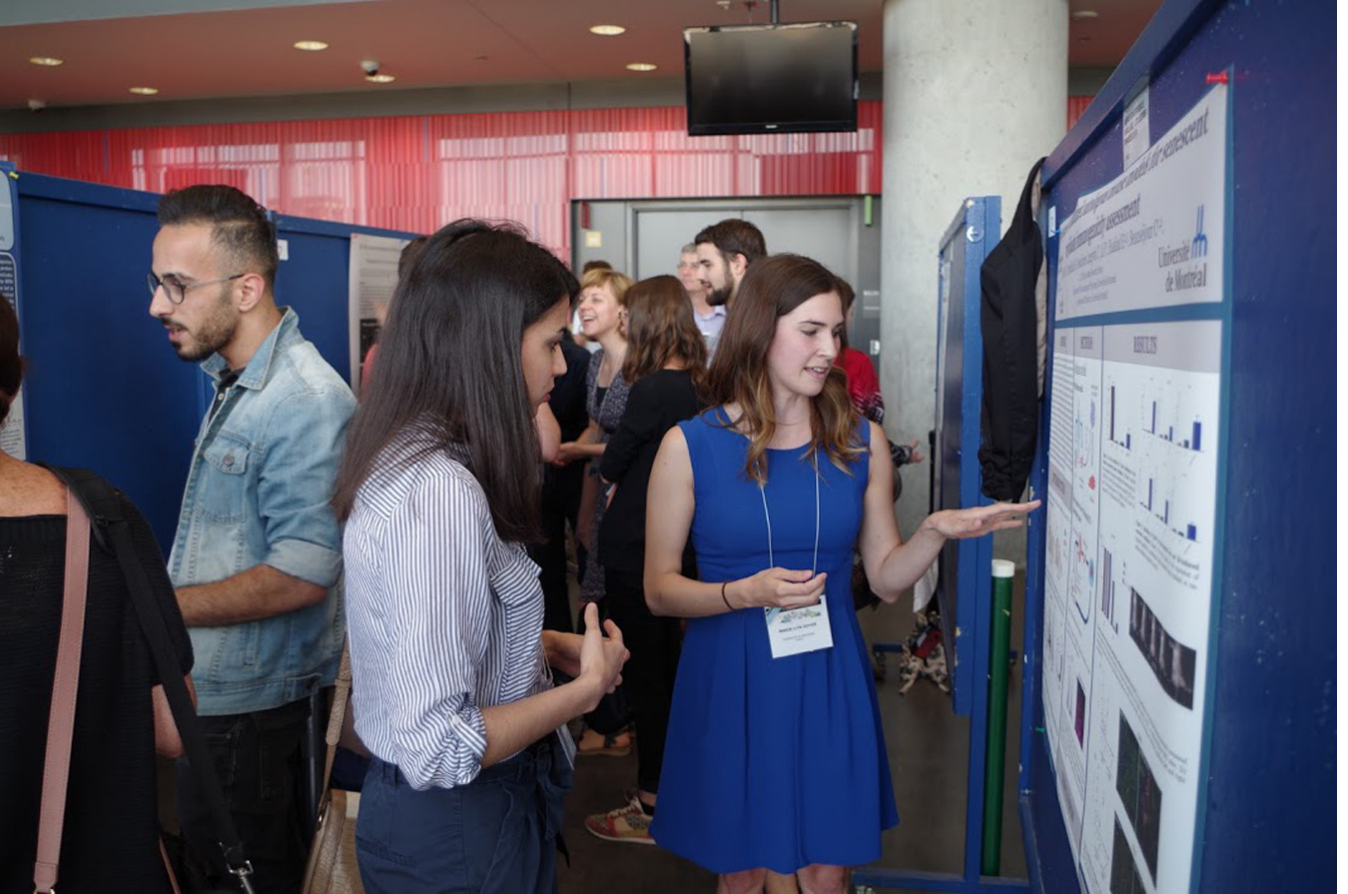
Poster Session Photo 8

**Figure:**
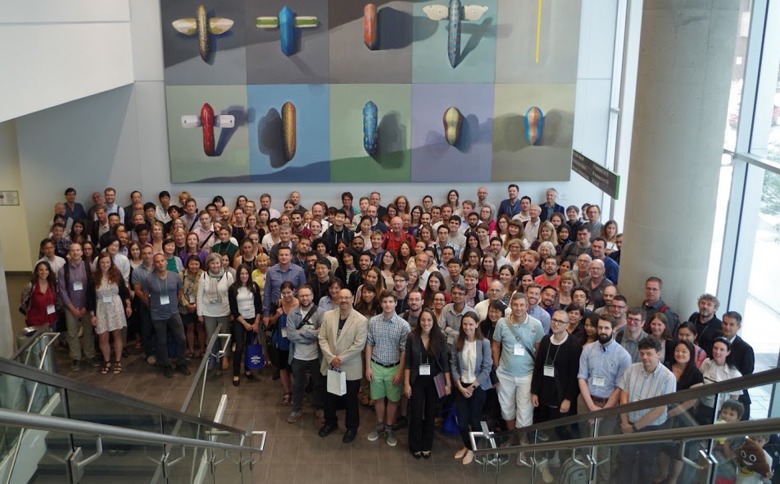
Group Photo
